# Impulsivity Moderates Skin Conductance Activity During Decision Making in a Modified Version of the Balloon Analog Risk Task

**DOI:** 10.3389/fnins.2019.00345

**Published:** 2019-04-15

**Authors:** Philippa Hüpen, Ute Habel, Frank Schneider, Joseph W. Kable, Lisa Wagels

**Affiliations:** ^1^Department of Psychiatry, Psychotherapy and Psychosomatics, School of Medicine, RWTH Aachen University, Aachen, Germany; ^2^JARA-Institute Brain Structure Function Relationship (INM 10), Research Center Jülich, Jülich, Germany; ^3^University Hospital Düsseldorf, Düsseldorf, Germany; ^4^Department of Psychology, University of Pennsylvania, Philadelphia, PA, United States

**Keywords:** decision making, risk, reward, skin conductance activity, impulsivity

## Abstract

Individual differences in traits such as impulsivity and processing of risk and reward have been linked to decision making and may underlie divergent decision making strategies. It is, however, unclear whether and how far individual differences in these characteristics jointly influence decision making. Here, we aimed to investigate the roles of skin conductance responses, a psychophysiological marker of risk processing and impulsivity, as assessed by the Barratt Impulsiveness Scale 11 on decision making. Forty-six healthy participants performed a modified version of the Balloon Analog Risk Task (BART), where reward and explosion risk are manipulated separately. Participants are informed about whether they play a high versus low reward and high versus low explosion risk condition. The exact risk and reward contingencies are, however, unknown to participants. Participants were less risk-taking under high, compared to low explosion risk and under high reward, compared to low reward on the modified BART, which served as a validation of the paradigm. Risk-taking was negatively related to skin conductance responses under high explosion risk. This relationship was primarily driven by individuals with relatively high levels of impulsivity. However, impulsivity alone was not found to be related to decision making on the modified BART. These results extend evidence that skin conductance responses may guide decision making in situations, where participants are informed about risk level (high vs. low), which might be differentially moderated by different levels of impulsivity.

## Introduction

Decisions under risk and uncertainty involve choosing among options that may be accompanied by a potential for negative outcome. Elucidating processes and individual differences associated with decision making under risk and reward is important, because detrimental decision making is involved in everyday life decisions (e.g., reckless driving, misinvestment in stocks), as well as in psychiatric disorders (e.g., borderline personality disorder, Attention-Deficit/Hyperactivity Disorder; Sebastian et al., 2013, 2014; Dekkers et al., 2016).

Previous research on risky decision making has highlighted individual differences in decision making strategies. In general, individuals tend to show a bias toward risk aversion in gain contexts. A small group of individuals, however, seems to show the opposite and prefers uncertain, large rewards over certain small ones (Kahneman and Tversky, 1979). Individual differences in subjective preferences typically guide decision makers’ (DMs) choices (Kable and Glimcher, 2007; Kóbor et al., 2015), and traits such as impulsivity and affective processing have been identified as important factors underlying decision making preferences. For example, various studies report associations between disadvantageous decision making and facets of impulsivity (Christodoulou et al., 2006; Bayard et al., 2011). Impulsivity is a multidimensional construct characterized by a predisposition toward rapid and unplanned actions without consideration of future consequences (Evenden, 1999). One facet of impulsivity that may be considered as an integral aspect of decision making is incomplete information sampling, in which the individual assesses the amount of available information before a decision is made. In this context, impulsivity can be considered as premature termination of information sampling prior to the decision (Stahl et al., 2014). Another way of assessing impulsivity is self-report measures. One of the most commonly used self-report measure is the Barratt Impulsiveness Scale 11 (BIS-11; Patton et al., 1995), which incorporates subscales (attentional impulsiveness, motor impulsiveness, non-planning impulsiveness), thereby accounting for the multifaceted nature of impulsivity. Premature termination of information sampling which might manifest as a tendency to make premature decisions and responses is captured by the BIS-11 attentional impulsiveness scale and the BIS-11 motor impulsiveness scale, respectively (Evenden, 1999; Caswell et al., 2015). However, self-reported impulsivity and laboratory assessments of impulsivity often yield different results (Reynolds et al., 2006), which may be due to its multifaceted nature. Another reason for the discrepancy may be the decision context, which may influence one’s susceptibility to impulsive actions by emphasizing rewards or punishments. Moreover, divergent evidence exists on the relation between trait impulsivity and decision making. Although previous research identified neural alterations related to decision making processes in impulsive individuals (Martin and Potts, 2009; Dinu-Biringer et al., 2016), behavioral evidence of increased risky decision making in these individuals is mixed. While some studies suggest that impulsive individuals show elevated levels of risky decisions (Franken et al., 2008; Sweitzer et al., 2008), others did not replicate such findings (Martin and Potts, 2009; Dinu-Biringer et al., 2016). The relationship between individual differences in trait impulsivity and decision making is, thus, poorly understood and further research is needed.

In addition to impulsivity, skin conductance responses (SCRs) are discussed in relation to decision making behavior (Bechara et al., 1997; Bechara and Damasio, 2005). They are thought to assess unconscious arousal by detecting changes in eccrine sweating, which is regulated by the autonomic nervous system. SCRs can be measured continuously and unobtrusively, providing a tracking method of affective processes involved in decision making. In fact, prior research has demonstrated the utility of SCRs as indicators of implicit risk attitudes (Bechara, 2003; Guillaume et al., 2009). An intriguing, well-replicated finding is that anticipatory SCRs are greater prior to making disadvantageous decisions, relative to advantageous ones (Bechara et al., 1997; Guillaume et al., 2009; Holper et al., 2014).

Research thus suggests that impulsivity and SCRs are linked to decision making. However, so far, the relationship between individual differences and decision making has been investigated mostly in isolation without considering a potential interplay between these different individual characteristics. Therefore, it is of interest to investigate whether and in how far individual differences in impulsivity and SCRs and their potential interplay influence decision making.

**FIGURE 1:**
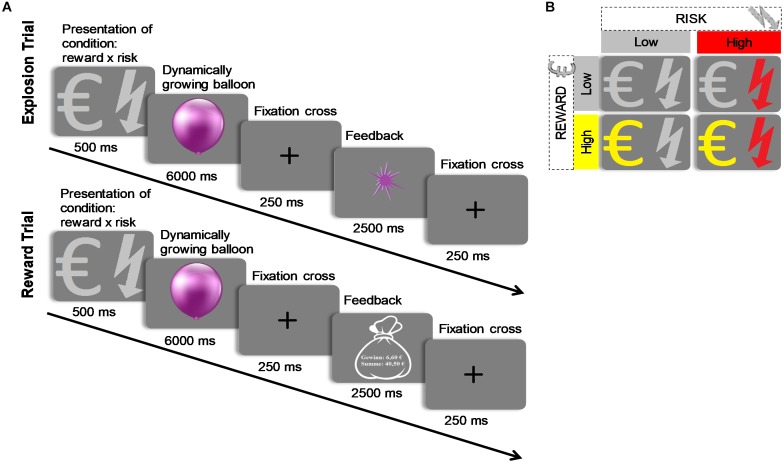
**(A)** Schematic of the modified BART. Participants were presented with a computerized balloon which is dynamically growing large. The increase in balloon size confers to greater risk of explosion, but also to greater potential reward. Participants determine the point of time at which the balloon should stop inflating and are informed about the outcome (explosion or reward) after a temporal delay. A potential explosion of the balloon is saved in the computer program, but is not visually presented to participants online. **(B)** Importantly, the modified BART employs a 2 × 2 design with two levels of risk (high vs. low) and two levels of reward (high vs. low). At the beginning of each trial, these conditions are presented to participants such that they know which condition they play.

Since different DMs seem to weigh the probability and magnitude of risk and reward differently (Quartz, 2009; Bland and Schaefer, 2012; Mather and Lighthall, 2012), it is important to consider these characteristics. Therefore, it has been emphasized to manipulate risk and reward separately when investigating cognitive and emotional processes related to decision making (Preuschoff et al., 2006). In the field of economics, risk is defined as the variance of possible outcomes, whereas in the field of psychology, risk-taking has a broader meaning. Examples may include driving under the influence of alcohol, having unprotected sex, or spending your salary on gambles. In all of these examples, risk refers to the increased probability of something “bad” happening. Despite the definition of risk in economics, which allows for a detailed decomposition into cognitive constructs such as magnitude of gains and losses and probability of outcomes, experimental paradigms sometimes have limited success predicting naturalistic risk-taking behavior. On the other hand, experimental paradigms developed by psychologists often correlate with naturalistic risk-taking behavior and induce affective arousal. However, generally, they do not allow for a careful decomposition of cognitive constructs. Therefore, it has been suggested to bridge the gap between economic and more naturalistic assessments of risk-taking (Schonberg et al., 2011). The Balloon Analog Risk Task (BART) is a prominent task assessing naturalistic risk-taking behavior, which entails participants sequentially pumping up a balloon by pressing a button (Lejuez et al., 2002). Each button press causes the balloon to inflate and money to be accrued up until some threshold, at which the balloon explodes. A larger balloon thus confers to greater reward but also to a greater probability of explosion. This nature of the task has the advantage that risk is dynamically increasing and one source of these changes in risk is well known – the explosion probability of the balloon. It is, however, not possible to differentiate between potential effects of explosion risk and those of reward on decisions as both increase proportionally with balloon size. To that end, we modified the BART by introducing a 2 × 2 design (see Figure 1) with reward (high vs. low) and explosion risk (high vs. low) which allows examining the relative effects of explosion risk and reward on decision making, while keeping the escalating tension of the BART, which is often intrinsic to naturalistic risk-taking. Critically, this manipulation of explosion risk increases risk in both the psychological sense, as there is a higher probability of bad outcomes, and in the economic sense, as the variance of the outcomes is greater within the typical range of behavioral responses in this task (see section Modified BART).

Moreover, in contrast to the original BART where participants sequentially inflate the balloon by button presses, in the modified version, the balloon automatically inflates and participants only press a response button to cash out. This enables us to track anticipatory SCRs related to decision making continuously. Therefore, assessing SCRs in the context of the modified BART with different decision making environments may provide an optimal tool for continually studying emotional correlates of decision making under risk and reward.

In the present study, we aim to (1) investigate the working mechanisms of the modified BART, and (2) examine (joint) effects of impulsivity and anticipatory SCRs on decision making under risk and reward.

## Materials and Methods

### Participants

A total of 46 (23 females, 23 males) individuals were recruited via public flyers and the RWTH Aachen University community. Participants met the following inclusion criteria: age between 18 and 50 years, high proficiency of the German language, no intake of medication affecting the central nervous system, no current substance abuse or addiction and no psychiatric or neurological diseases.

### Procedure

After giving informed consent, participants were seated in a laboratory room of the RWTH Aachen University Hospital. The laboratory consisted of two rooms that were connected by a passage. Participants were seated in one room, while the experimenter remained in the connected second room during study completion. After completing an intake survey (demographic and self-report questionnaires), participants were prepared for the SC measurement. Specifically, two electrodes were placed on the medial phalanges of the index and middle finger of the non-dominant hand. Subsequently, participants played four trials of the modified BART in order to practice, to let the experimenter check the SC signal, and to ensure a good and stable electrical connection between electrodes and skin. Participants were told to comfortably rest their hand on the desk and to avoid any movements. Finally, participants performed the modified BART which lasted about 25 min.

### Measures

#### Modified BART

In the modified BART, a dynamically growing balloon is presented for 6000 ms (see Figure 1). The increase in balloon size confers greater risk of an explosion, but also greater potential reward. Participants indicate by a button click when they want to cash out. Importantly, even after participants decide to cash-out and press the response button, the balloon keeps on growing larger until 6000 ms have elapsed. The explosion of the balloon is, thus, not visible to participants online but only occurs in the background of the computer program. This way, all trials can be analyzed, which avoids potential truncation of the data. Such a response structure where participants make one choice out of a multitude of possible choices and receive delayed feedback enables performance which is not affected by possible violations of the reduction axiom (Crosetto and Filippin, 2013; Filippin and Crosetto, 2016). In contrast, estimates of risk attitudes based on the original BART, usually, include only trials on which balloons did not explode. The balloon presentation is followed by a fixation cross (250 ms) and a feedback phase (2500 ms). In case participants respond before the balloon explodes in the background of the program, positive feedback is presented. Positive feedback is indicated by a moneybag presenting the amount of money won in that specific trial and the total amount accrued over all trials. In case participants respond after the balloon explodes in the background of the program, negative feedback (explosion sign) is presented. Response time (RT) is the variable indexing risky decision making.

The modified BART employs a 2 × 2 design with two levels of risk (high vs. low) and two levels of reward (high vs. low). At the beginning of each trial, these conditions are presented (500 ms) participants know which condition they play.

In order to establish the illusion of an inflating balloon, 128 pictures of balloons in increasing size are presented. A (cumulative) probability of explosion was arranged by assigning a probability value to each of the 128 pictures. For the low risk conditions, the probability of explosion associated with the first picture is 1/128, the probability of explosion associated with the second picture is 2/128 and so on up until the 128th picture, at which the probability of an explosion is 128/128. At the time of the participant’s response, a random number is drawn and compared against the explosion probability assigned to the picture at which the participant responded determining outcome (balloon explosion or reward). The probability of explosion on high risk trials is 1.5 times greater compared to low risk trials for balloon pictures 1 to 84. Thus, the probability of explosion is 1/128^∗^ 1.5 = 0.0117 for the first balloon picture of high risk trials. Explosion probability on high risk trials for balloon pictures 85 to 127 is 0.99 and 1 for balloon picture 128. Reward is also arranged by assigning a reward value (Euro cents) to each of the 128 balloon pictures. If the participant cashes out on the first balloon picture and the balloon does not explode, five cents are awarded to the participant on the low risk condition. The reward increases linearly by the factor of five. On the high risk condition, reward is five times greater compared to the low risk condition. Thus, reward is 5^∗^5 = 25 cents for the first balloon picture on high risk trials and increases linearly by the factor of 25. It should be noted that the variance of potential outcomes, or risk in economic terms, is also increased by increasing reward sizes. Therefore, the variance of outcomes differs across all four conditions and can take greatest values in the high risk/high reward condition.

The modified BART encompasses 40 trials per condition, thus 160 trials in total. Conditions were presented in a pseudorandom order. The sequence, determined by an algorithm was, thus, identical for every participant. It was not allowed that the same condition occurred three times in a row. The paradigm was programmed and presented using the Presentation^®^ software of neurobehavioral systems^1^.

#### SCRs

Exosomatic SC was measured with a direct current using Brain Vision Recorder (Brain Products GmbH, Gilching, Germany) while participants performed the modified BART. Two grounded flat silver/silver chloride (Ag-AgCl) electrodes of 10 mm diameter were placed at the distal phalanges of the index and middle fingers of the non-dominant hand. In order to optimize current flow, electrodes were prepared with a 0.5% saline paste in a neutral base (Med Associates TD-246). SC data were recorded at 5000 Hz and a direct current excitation voltage of 0.5 V. The recording was synchronized with the modified BART task sequence via condition-specific triggers send by the Presentation^®^ software (see footnote 2).

Data was preprocessed with BrainVision Analyzer. First, sampling rate was changed to 80 Hz. Afterward, data were segmented according to the four conditions (i.e., risk × reward) and exported to Ledalab. Further analyses were performed using the Ledalab toolbox (V.3.4.8) based on standardized procedures as recommended, including smoothing using the Gauss-method and a window width of 16 samples and data filtering applying a low-pass Butterworth filter with a cutoff frequency of 2 Hz (Benedek and Kaernbach, 2010). Subsequently, a continuous decomposition analysis (CDA) was performed since SC raw data are characterized by phasic skin conductance responses overlying a tonic component which can be separated by CDA (Benedek and Kaernbach, 2010). This analysis method follows four steps: estimation of a parameter describing tonic activity (1), non-negative deconvolution of phasic SC data resulting in a driver function and a non-negative remainder (2), segmentation of driver and remainder identifying single impulses by peak detection (3), reconstruction of SC data (4).

Since our aim was to assess anticipatory SCRs underlying the time course of decisions in response to risk and reward (i.e., the time during which the balloon inflated), we took the time integral of the phasic driver over the entire response window as our dependent variable. The phasic driver time integral reflects the cumulative phasic activity (Benedek and Kaernbach, 2010). The response window was 1–6 s after condition presentation (i.e., risk × reward). For peak detection, a minimum amplitude criterion of 0.05 μS was used.

#### BIS-11

Trait impulsivity was assessed by means of the BIS-11 (Patton et al., 1995; Stanford et al., 2009). It is the most widely used self-report instrument for the assessment of trait impulsiveness and comprises 30 items, which are to be rated on a 4-point Likert scale reflecting the frequency of occurrence. We took the total score over all 30 items (after reversing scores for appropriate items) as a participant’s impulsivity score, which is recommended for the German version (Preuss et al., 2008).

### Statistical Analyses

#### Preliminary Analysis –Effectiveness of the Modified BART in Assessing Decision Making

In order to examine the effectiveness of the modified BART in assessing risky decision making and to investigate the effect of different levels of risk and reward on decision making, we performed a repeated measures Multivariate Analyses of Variance (MANOVA) with two within-subjects factors, having two levels each: risk (high vs. low) and reward (high vs. low). Dependent variables were (1) response time (RT), (2) successful trials (i.e., trials on which the balloon did not explode), and (3) earnings. Our primary dependent variable of interest was RT, the index of risky decision making on the modified BART. However, for a comprehensive overview of the consequences of participants’ decisions, we also examined number of successful trials and earnings. Significant multivariate effects were followed-up by univariate ANOVAs which were, in turn, followed by Bonferroni-corrected *post hoc* *t*-tests. Cohen’s *d* was calculated for significant mean differences as an index of the size of an effect. According to Cohen’s conventional guidelines effect sizes of 0.20 ≤*d* ≤ 0.50 are considered as small, whereas effect sizes of 0.50 ≤*d* ≤ 0.80 and *d* ≥ 0.80 are considered as moderate and large size, respectively (Cohen, 1977).

#### Main Analysis –Effects of Impulsivity and SCRs on Decision Making

Since we had repeated measures within participants and a relatively small sample, we fitted a linear mixed-effects model with random intercepts for participants and used restricted maximum likelihood for the estimation of variance components using the R (R Core Team, 2014) package “lme4” (Bates et al., 2015). Restricted maximum likelihood estimation of variance components account for lost degrees of freedom resulting from testing fixed effects parameters (Harville, 1977). The dependent variable was RT and fixed effects were the repeated factors risk and reward, and the covariates BIS-11 impulsivity scores and SCRs. Since we were specifically interested in potential interacting effects of BIS-11 scores and SCRs, we also included interaction terms of these covariates for the different conditions. In order to disentangle significant interactions including continuous predictors, exploratory simple slope analyses using the Johnson-Neyman interval were performed (Long, 2018).

## Results

### Effectiveness of the Modified BART in Assessing Decision Making

**FIGURE 2:**
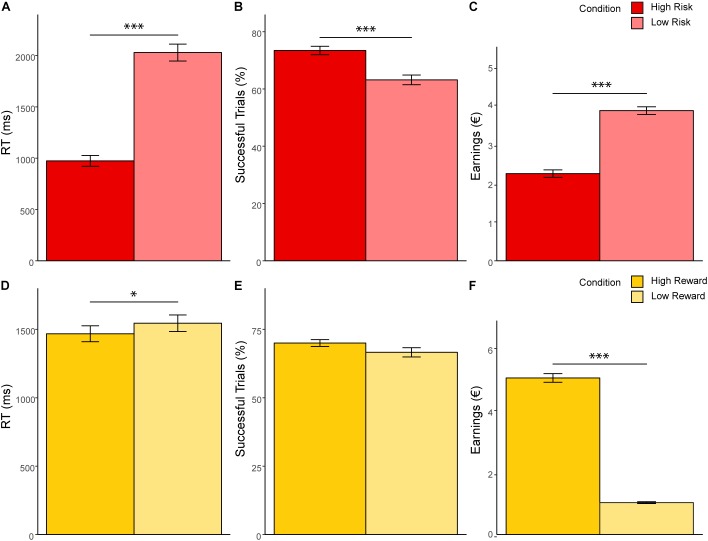
MANOVA results. **(A–C)** show response time, percentage of balloons which did not explode and earnings as a function of risk level. **(D–F)** show response time, percentage of balloons which did not explode and earnings as a function of reward level. Error bars represent standard errors of the means. ^∗^*p* < 0.05. ^∗∗∗^*p* < 0.001.

**FIGURE 3:**
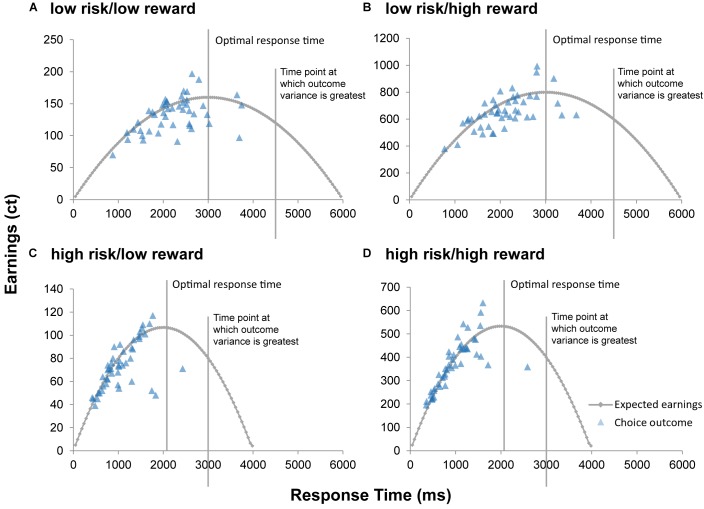
Expected value of cash outs. The curve represents the expected value of cash outs for each time point of the four conditions **(A–D)** of the modified BART. Optimal response time (time point at which the expected value of cash outs is greatest) and time point at which variance of outcomes is greatest are indicated for each condition. Optimal response time for the low risk/low reward condition **(A)** and for the low risk/high reward condition **(B)** is 3,000 ms. Optimal response time for the high risk/low reward condition **(C)** and for the high risk/high reward condition **(D)** is 2,015 ms. For each participant, mean earnings (in euro cents) per condition is mapped as a function of response time.

Significant results of the repeated measures MANOVA indicated a linear combination of the dependent variables RT, successful trials, and earnings for which the different levels of risk [Pillais’ Trace = 0.85, *F*(3,39) = 75.67, *p* < 0.001, η_p_^2^ = 0.853], reward [Pillais’ Trace = 0.97, *F*(3,39) = 433.31, *p* < 0.001, η_p_^2^ = 0.971], and the interaction effect of risk and reward [Pillais’ Trace = 0.83, *F*(3,39) = 64.38, *p* < 0.001, η_p_^2^ = 0.832] differed. Follow-up univariate ANOVAs revealed statistically significant main effects of risk on RT [*F*(1,41) = 170.89, *p* <0.001, η_p_^2^ = 0.807], successful trials [*F*(1,41) = 46.20, *p* < 0.001, η_p_^2^ = 0.530], and earnings [*F*(1,41) = 213.37, *p* < 0.001, η_p_^2^ = 0.839]; statistically significant main effects of reward on RT [*F*(1,41) = 4.97, *p* = 0.031, η_p_^2^ = 0.108], and earnings [*F*(1,41) = 1011.36, *p* < 0.001, η_p_^2^ = 0.961], but not on successful trials [*F*(1,41) = 4.01, *p* = 0.052, η_p_^2^ = 0.089]; and a statistically significant interaction effect of risk and reward on earnings [*F*(1,41) = 134.40, *p* < 0.001, η_p_^2^ = 0.776], but not on RT [*F*(1,41) = 1.12, *p* = 0.306, η_p_^2^ = 0.026] and successful trials [*F*(1,41) = 0.96, *p* = 0.33, η_p_^2^ = 0.013]. Bonferroni-corrected *post hoc* tests regarding the main effect of risk indicated that RT was faster for high risk (*M* = 973.59, *SD* = 52.86) compared to low risk (*M* = 2028.99, *SD* = 82.28) trials (*p* < 0.001, *d* = 15.41), participants had more successful trials for high risk (*M* = 0.75, *SD* = 0.01) compared to low risk (*M* = 0.65, *SD* = 0.02) trials (*p* < 0.001, *d* = 7.89), and earnings were lower for high risk (*M* = 2.24, *SD* = 0.09) compared to low risk (*M* = 3.84, *SD* = 0.10) trials (*p* < 0.001, *d* = 15.99; see Figure 2). With regard to the main effect of reward, *post hoc* tests revealed that RT was shorter for high reward (*M* = 1467.76, *SD* = 58.21) as compared to low reward (*M* = 1544.82, *SD* = 60.66) trials (*p* < 0.001, *d* = 1.47), and that earnings were greater for high reward (*M* = 5.05, *SD* = 0.14) as compared to low reward (*M* = 1.03, *SD* = 0.03) trials (*p* < 0.001, *d* = 39.08; see Figure 2). The finding that earnings were greater for high as compared to low reward conditions and greater for low as compared to high risk conditions lies within the nature of the task and will not be further discussed. Consequently, also the interaction of risk and reward on earnings was significant, revealing an ordinal interaction, where the differences in earnings for different levels of reward (*M* difference = 5.05, *SD* = 0.956) were dependent on levels of risk (*M*_difference_ = 2.99, *SD* = 1.03), *t*(41) = 11.91, *p* < 0.001, *d* = 2.07. Figure 3 depicts participants’ risk-taking behavior in relation to the four different conditions.

### Main Analysis –Effects of Impulsivity and SCRs on Decision Making

All parameter estimates for the fixed effects with RT as the dependent variable are presented in Table 1. Summary statements regarding significant fixed effects are reported below. Variance and standard deviation for the intercept of the random effect (participant) were 194,103, and 440.6, respectively.

**Table 1:** Parameter estimates from the linear mixed model analysis of risk, reward, impulsivity, skin conductance responses, and corresponding interaction terms on response times.

	*b*	SE	*t*	95% CI
Intercept	156.341	871.911	0.18	–1566.98 to 1829.45
Reward	414.128	637.274	0.65	–801.00 to 1634.58
SCR	1309.113	932.037	1.41	–504.37 to 3150.34
BIS-11	16.412	14.913	1.10	–12.27 to 46.01
Risk	1888.034	665.497	2.84*	619.85 to 3163.47
Reward × SCR	–136.666	819.115	–0.17	–1707.90 to 1424.12
Reward × BIS-11	–4.552	10.941	–0.42	–25.51 to 16.3072
SCR × BIS-11	–25.776	15.575	–1.66	–56.76 to 4.69
Risk × SCR	–2228.632	903.361	–2.47*	–3969.54 to -506.66
Risk × BIS-11	–14.248	11.501	–1.24	–36.30 to 7.66
Reward × SCR × BIS-11	0.970	13.708	0.07	–25.15 to 27.28
Risk × SCR × BIS-11	39.635	15.442	32.57*	10.18 to 69.44


*Linear mixed model fit by restricted maximum likelihood estimation. SE, Standard Error; CI, confidence interval; SCR, skin conductance response; BIS, Barratt Impulsiveness Scale. ^∗^*p* < 05 (*t*-tests are based on Satterthwaite’s degrees of freedom method).*

**FIGURE 4:**
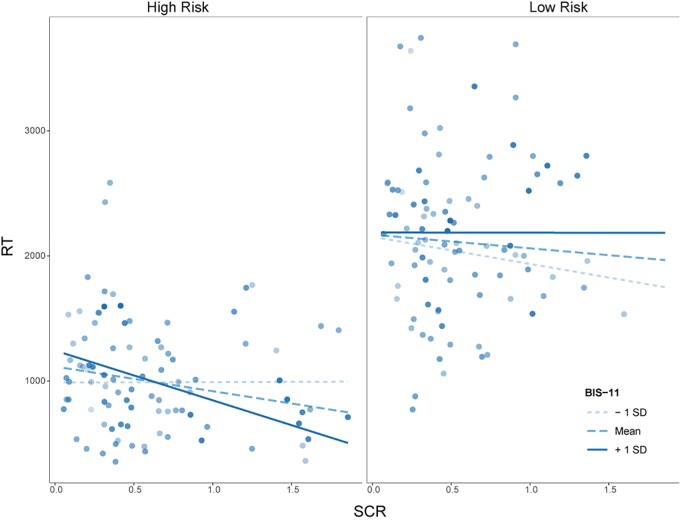
Simple slopes for the interaction of skin conductance response (SCR) and Barratt Impulsiveness Scale 11 (BIS-11) scores at mean ± 1 *SD* for high and low risk. Circles represent observed data on participants’ response times (RTs) as a function of SCR.

A main effect of risk [*t*(130.49) = 2.84, *p* < 0.01] demonstrated that RT was longer under low risk than under high risk, as also revealed by the MANOVA reported above. Furthermore, the interaction between SCRs and risk was significantly related to RT, *t*(143.27) = 2.47, *p* < 0.014. Associated *post hoc* tests revealed a negative relationship between SCRs and RT only under high risk (see Table 2). Our main interest regarding this mixed model analysis lied in a potential interaction of SCRs, impulsivity and risk or reward. The interaction between SCRs, impulsivity and reward was not significant, *t*(127.97) = 0.07, *p* = 0.94. However, the interaction between SCRs, impulsivity and risk was significantly related to RT, *t*(145.71) = 2.57, *p* < 0.011. *Post hoc* tests revealed that under high risk, greater SCRs were related to lower RTs for individuals with high levels of impulsivity (see Figure 4 and Table 2).

**Table 2:** Exploratory *post hoc* tests on simple slopes for interaction terms.

Significant interaction effects for the mixed model	Simple slopes models		*b*	SE	df	*p-*value
Risk × SCR	Slope for SCR when risk = low		–149.66	173.91	–0.86	0.39
	Slope for SCR when risk = high		–237.07	132.50	–1.79	<0.05
Risk × SCR × BIS-11	Risk = low	Slope for SCR when BIS = 50.64 (-1 *SD*)	–261.46	227.90	–1.15	0.25
		Slope for SCR when BIS = 58.43 (Mean)	–149.66	173.91	–0.86	0.39
		Slope for SCR when BIS = 66.23 (+ 1 *SD*)	–37.86	245.27	–0.15	0.88
	Risk = high	Slope for SCR when BIS = 50.64 (- 1 *SD*)	–39.95	184.92	–0.22	0.83
		Slope for SCR when BIS = 58.43 (Mean)	–237.07	132.50	–1.79	0.08
		Slope for SCR when BIS = 66.23 (+ 1 *SD*)	–437.19	166.79	–2.60	<0.05


*SE, standard error; df, degrees of freedom; SCR, skin conductance responses; BIS, Barratt Impulsiveness Scale; *SD*, standard deviation.*

## Discussion

Balancing magnitude and probability of risk and reward is crucially involved in successful decision making. Although the original BART has been found to reliably assess risk-taking attitudes, it does not allow a careful differentiation between these two factors. Here, we present results of a modified version of the BART, which is designed to investigate the effects of different levels of risk and reward on decision making.

We found that during the modified BART, DMs took fewer risks under high risk compared to low risk as evidenced by fewer balloon explosions and faster RTs. This finding suggests that, in general, DMs were cautious when placed in high risk situations. We also show that DMs exhibited less willingness to take risks when high rewards were at stake. Since higher rewards also increase the variance of outcomes, our results can be explained by economic descriptions of risk aversion. Variance of outcomes increase monotonically with cumulative explosion probability, in this task up 75% and most participants stop well before (see Figure 3). Summarized, the overall pattern of our results is consistent with previous findings that the majority of individuals are risk aversive when gains – especially high gains – are at stake (Kahneman and Tversky, 1979; Christopoulos et al., 2009). Here, we show the importance of considering both risk and reward and validate that the modified BART may be a useful measure to assess risky decision making.

Our main analyses included investigating the role of SCRs and impulsivity on decision making under risk and reward. We found a negative relationship between SCRs and RTs when explosion risk was high. Previous research has already demonstrated that individuals exhibit greater anticipatory SCRs before making risky decisions, compared to relatively safe ones (Bechara et al., 1997; Crone et al., 2004; Guillaume et al., 2009). In this line, it has been suggested that SCRs may guide decision making processes. These findings primarily stem from studies using the Iowa Gambling Task (IGT), which requires learning different reward and punishment contingencies. However, some studies have extended these findings of enlarged SCRs preceding risky choices for tasks which do not require learning (i.e., where risk and reward contingencies are explicit). Our results further extend these findings in so far that greater SCRs seem to be associated with reduced risk-taking behavior when individuals are placed in high risk situations. This finding adds evidence to the notion that SCRs may guide risky decision making.

Importantly, the negative relationship between SCRs and RTs was especially driven by individuals with relatively high levels of impulsivity. These individuals took fewer risks, as indexed by shorter RTs when they also had greater SCRs, while they took more risks when they had lower anticipatory SCRs. On the low risk condition, there was no such relationship present (i.e., no relation between SCRs and RT). It may be the case that this small group of individuals with high impulsivity scores and high anticipatory SCRs was more sensitive to explosion risk, as observed in their psychophysiological responding and modulated their behavior accordingly. In contrast, individuals with low trait impulsivity who exhibited high anticipatory SCRs, had higher RTs in high risk, but lower RTs in low risk situations. However, statistical significance of exploratory *post hoc* tests for this effect is missing and it has to be further investigated whether impulsivity actually inverts the relationship of SCRs and risk-taking under low risk.

Previous findings on the relationship between impulsivity and decision making are divergent, with some studies reporting detrimental effects of impulsivity on decision making (Franken et al., 2008; Sweitzer et al., 2008), while others could not report any associations (Martin and Potts, 2009; Dinu-Biringer et al., 2016). In contrast, we found that higher levels of impulsivity may beneficially alleviate risk-taking behavior. Therefore, impulsivity might not always be detrimental to decision making as some studies suggest (Christodoulou et al., 2006; Franken et al., 2008; Sweitzer et al., 2008). In fact, some studies have illustrated that trait impulsivity may be functional under certain circumstances (Dickman, 1990), including decision making (Burnett Heyes et al., 2012). It should be noted that impulsivity scores in our sample, were still within the normal range (c.f., Preuss et al., 2008). In order to gain a more comprehensive overview of the dimensionality of impulsivity, further investigation is needed on whether decision making deficits are observed in individuals with extreme impulsivity scores and in impulsive patient groups (Dinu-Biringer et al., 2016). It should be stressed that we could not find evidence for impulsivity alone to be related to risky decision making. A meta-analysis on the relation between impulsivity and decision making on the original BART also found negligible to small effects (Lauriola et al., 2014). In fact, it has been suggested that previously reported links between impulsivity and risky decision making may actually be related to confounding factors (Bayard et al., 2011). One of these confounds, or moderators, may be SCRs, indices of arousal processes in anticipation of decisions under risk (Bechara and Damasio, 2005).

In conclusion, our results support the notion that anticipatory SCRs guide decision making processes and suggest that elevated SCRs under high explosion risk may be related to less risk-taking behavior. This negative relationship between SCRs and risky decision making was particularly driven by individuals with relatively greater levels of trait impulsivity, which might indicate greater sensitivity to explosion risk and stronger modulation of behavior. Our findings suggest that impulsivity may not always be detrimental to decision making and may explain divergent previous findings on the relationship between impulsivity and risky decision making in so far that risk processing and impulsivity seem to interactively influence decision making. Further research should be conducted to replicate these results, possibly in a larger sample with a wider range of impulsivity scores and to explore these relationships in clinical populations.

## Ethics Statement

The study was approved by the Ethics Committee of the Medical Faculty of the RWTH Aachen University, all participants gave oral and written informed consent, and no adverse events occurred. Participants were paid 20.00 Euros as financial reimbursement for study participation.

## Author Contributions

PH, UH, FS, JK, and LW contributed to the conception and design of the study. PH and LW acquired the data and performed data analyses. PH organized the database. PH, UH, and LW contributed to data interpretation. PH wrote the first draft of the manuscript. All authors read and approved the submitted version.

## Conflict of Interest Statement

The authors declare that the research was conducted in the absence of any commercial or financial relationships that could be construed as a potential conflict of interest.

## Footnotes

1 www.neurobs.com
